# Implementing school nursing strategies to reduce LGBTQ adolescent suicide: a randomized cluster trial study protocol

**DOI:** 10.1186/s13012-016-0507-2

**Published:** 2016-10-22

**Authors:** Cathleen E. Willging, Amy E. Green, Mary M. Ramos

**Affiliations:** 1 Behavioral Health Research Center of the Southwest, Pacific Institute for Research and Evaluation, 612 Encino Place NE, Albuquerque, NM USA; 2 Department of Psychiatry, University of California, San Diego, 9500 Gilman Drive, MC 0812, La Jolla, San Diego, CA 92093 USA; 3 Child and Adolescent Services Research Center, 3665 Kearny Villa Road, San Diego, CA 92123 USA; 4 Department of Pediatrics, University of New Mexico, MSC10 5590, 1 University of New Mexico, Albuquerque, NM 87131-0001 USA

**Keywords:** Evidence-based practice, Implementation, Sexual and gender minority, School nurses—suicide

## Abstract

**Background:**

Reducing youth suicide in the United States (U.S.) is a national public health priority, and lesbian, gay, bisexual, transgender, and queer or questioning (LGBTQ) youth are at elevated risk. The Centers for Disease Control and Prevention (CDC) endorses six evidence-based (EB) strategies that center on meeting the needs of LGBTQ youth in schools; however, fewer than 6 % of U.S. schools implement all of them. The proposed intervention model, “RLAS” (Implementing School Nursing Strategies to Reduce LGBTQ Adolescent Suicide), builds on the Exploration, Preparation, Implementation, and Sustainment (EPIS) conceptual framework and the Dynamic Adaptation Process (DAP) to implement EB strategies in U.S. high schools. The DAP accounts for the multilevel context of school settings and uses Implementation Resource Teams (IRTs) to facilitate appropriate expertise, advise on acceptable adaptations, and provide data feedback to make schools implementation ready and prepared to sustain changes.

**Methods/Design:**

Mixed methods will be used to examine individual, school, and community factors influencing both implementation process and youth outcomes. A cluster randomized controlled trial will assess whether LGBTQ students and their peers in RLAS intervention schools (*n* = 20) report reductions in suicidality, depression, substance use, bullying, and truancy related to safety concerns compared to those in usual care schools (*n* = 20). Implementation progress and fidelity for each EB strategy in RLAS intervention schools will be examined using a modified version of the *Stages of Implementation Completion *checklist. During the implementation and sustainment phases, annual focus groups will be conducted with the 20 IRTs to document their experiences identifying and advancing adaptation supports to facilitate use of EB strategies and their perceptions of the DAP.

**Discussion:**

The DAP represents a data-informed, collaborative, multiple stakeholder approach to progress from exploration to sustainment and obtain fidelity during the implementation of EB strategies in school settings. This study is designed to address the real-world implications of enabling the use of EB strategies by school nurses with the goal of decreasing suicide and youth risk behaviors among LGBTQ youth. Through its participatory processes to refine and sustain EB strategies in high schools, the RLAS represents a novel contribution to implementation science.

**Trial registration:**

ClinicalTrials.gov, NCT02875535

**Electronic supplementary material:**

The online version of this article (doi:10.1186/s13012-016-0507-2) contains supplementary material, which is available to authorized users.

## Background

In 2012, the United States (U.S.) Surgeon General identified lesbian, gay, bisexual, transgender, and queer or questioning (LGBTQ) youth as at heightened risk for suicide [1]. Risk factors include depression, substance use, inadequate social support, and not feeling safe at school [1–7]. Large population-based studies over the past 15 years have found that lesbian, gay, and bisexual (LGB) youth are at two to four times increased risk for suicidal ideation and attempts when compared to their cisgender, heterosexual peers [1–3, 5, 8–16]. Within LGB populations, suicidality disproportionately affects racial and ethnic minorities, including American Indian and Hispanic people [1, 17, 18]. In numerous surveys and qualitative studies, transgender youth report elevated risk for suicide, depression, and substance use [19–24].

In general, LGBTQ youth report high levels of rejection, harassment, victimization, violence, and sexual abuse that can contribute to mental health problems and suicide behaviors [3, 9, 13, 18, 25]. These behaviors relate to “minority stress,” i.e., chronic stress from stigmatization, prejudice, and discrimination [26]. National school climate surveys have found that LGBTQ youth are often exposed to minority stress in schools [25, 27]. Youth describe being victimized because of their known, or perceived, sexual orientation or gender identity [8–11, 13, 19, 27]. LGBTQ youth are also more likely to experience high school victimization when they disclose their orientation, self-identify as a sexual minority, recognize same-sex feelings at a younger age, or demonstrate gender-atypical behavior [14, 28].

LGBTQ youth with greater school connectedness and safety report lower suicidal ideation and attempts [29]. Gay-Straight Alliances (GSAs)—peer-to-peer support groups—protect against suicide and depression for LGBTQ students [25, 30–34]. School policies are also pivotal to the mental health of LGBTQ youth. LGBTQ students at schools with anti-harassment policies may feel safer and are less likely to be harassed [35]. Those at schools with supportive staff, anti-bullying policies, and GSA clubs are less likely to be victimized, skip school because of safety concerns, or attempt suicide compared with those in other schools [32]. LGBTQ youth may be at lower risk for attempting suicide if they attend school in districts with anti-bullying policies covering sexual orientation and gender identity [36]. LGBTQ students in settings with more protective school climates report fewer suicidal thoughts than those in places with less protective climates [37].

A supportive, safe school environment is key to a comprehensive public health strategy to prevent youth suicide [38]. As a protective factor against suicidal ideation and attempts, school connectedness is second in importance only to family connectedness [39]. The Centers for Disease Control and Prevention (CDC) endorses six EB strategies for schools to meet the needs of LGBTQ youth (see Table 1) [40]. However, the 2012 School Health Profiles Report based on data from 44 states found that only 5.5 % of secondary schools implement all six [40].

**Table 1:** Description of the school-based evidence-based strategies to meet the needs of LGBTQ youth

1. Identify “safe spaces” such as a counselor’s office, designated classroom, or student organization where LGBTQ youth can receive support from administrators, teachers, other school staff, or other students.
2. Prohibit harassment and bullying based on a student’s perceived or actual sexual orientation or gender expression.
3. Facilitate access to providers not on school property who have experience delivering health services, including human immunodeficiency virus (HIV)/sexually transmitted infection (STI) testing, counseling, and reproductive healthcare, to LGBTQ youth.
4. Facilitate access to providers not on school property who have experience in providing social and psychological services to LGBTQ youth.
5. Encourage staff members to attend professional development on safe and supportive school environments for all students, regardless of sexual orientation, gender identity, or gender expression.
6. Provide health education curricula or supplemental materials, i.e., HIV, STI, or pregnancy prevention information relevant, to LGBTQ youth (e.g., curricula or materials that use inclusive language or terminology).

Several factors within an organization can impact the success of implementation efforts. For example, organizational culture and climate influence staff willingness to engage in new practices [41], as do job tenure and level of professional development [42]. Leadership is also important [43]. Implementation leaders require capacity to be successful change agents and local champions, and their ability to motivate and interact effectively shapes staff attitudes toward adopting an EB strategy [44]. Personal innovativeness or ability to adapt or change can also impact attitudes toward new ways of working in a team or organization [45]. Provider attitudes toward adopting EB practices are also associated with actual utilization [46].

Schools are vital but largely untapped venues for intervention research on LGBTQ youth [2]. However, efforts to address the unmet mental health needs of youth in general and LGBTQ students specifically may not reach their full potential due to implementation challenges in school settings. The proposed intervention model, “RLAS” (Implementing School Nursing Strategies to Reduce LGBTQ Adolescent Suicide), builds on the Exploration, Preparation, Implementation, and Sustainment (EPIS) conceptual framework and the Dynamic Adaptation Process (DAP) to implement EB strategies in U.S. high schools [47, 48].

**Fig. 1:**
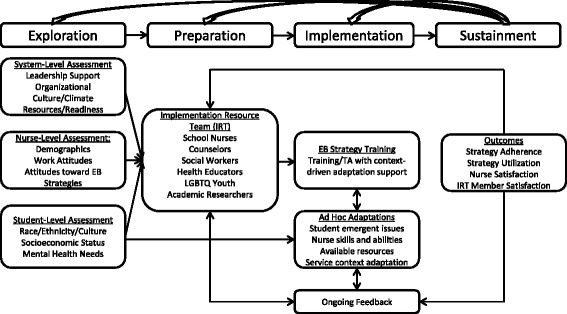
Key Dynamic Adaptation Process components to support evidence-based strategy implementation per the Exploration, Preparation, Implementation, and Sustainment framework

The EPIS framework segments implementation into four phases: *exploration* (considering new approaches to carry out EB strategies); *preparation* (planning to apply EB strategies); *implementation* (ongoing planning, training, coaching, and use of EB strategies); and *sustainment* (maintaining EB strategies over time) [47]. Per Fig. 1, the model emphasizes three levels of influence: system (or school), provider (or school nurse), and client (LGBTQ student). The model also attends to factors pertinent to both the “inner context” (e.g., schools, school nurses) and the larger “outer context” (e.g., policies, funding, resources) of EB strategy implementation and sustainment.

The DAP is an implementation strategy guided by the EPIS framework that provides direction for activities to undertake during each EPIS phase and a continuously iterative data-informed approach to support EB strategy implementation [48]. The DAP has four key components: initial assessment; stakeholder engagement and training; problem solving; and outcomes feedback to address challenges. Another core feature of the DAP is the development of an Implementation Resource Team (IRT), a collaboration comprised of multiple stakeholders to assist with implementation, interpret data, and address adaptation as an explicit part of the implementation process.

Interventions to promote mental health in schools often fail because they want for leadership and staffing [49]. Leaders can positively or negatively impact the capacity to foster change and thus are instrumental in facilitating a positive climate and attitudes for implementing EB strategies [43, 44]. The Institute of Medicine has recommended that nurses in the U.S. should “practice to the full extent of their education and training” and be “full partners” in “redesigning healthcare” [50]. In the current study, school nursing expertise is applied in a distinctive way to lead IRTs in implementing the six EB strategies in their schools. This study combines qualitative methods (e.g., in-depth interviews and focus groups) with population-based surveys and other quantitative measures to pursue three specific aims:

1. Utilize the DAP to enable specially-trained school nurse champions and IRTs to implement and sustain EB strategies to address the needs of LGBTQ high school students.
2. Conduct a cluster randomized controlled trial to assess whether LGB youth and peers in RLAS intervention schools report reduced suicidality, depression, substance use, and bullying, and increased safety compared to control schools.
3. Examine the individual, school, and community factors influencing implementation and outcomes.

## Methods/Design

### Study context

This work will occur in New Mexico (NM) high schools. Sociodemographic and minority stress factors contributing to adverse psychosocial outcomes are common in NM, which ranks 43rd in personal income per capita ($34,133) and second in persons below the poverty level (20.2 %) [51]. Of NM’s 33 counties, 32 are medically underserved [52]. Suicide rates for NM youth, ages 15 to 24, are nearly twice the national rate (19.7/100,000) [53, 54]. American Indian and Hispanic people comprise almost 56 % of 2,085,287 NM residents [55] and, like LGBTQ people, are disproportionately affected by health disparities [56, 57].

The New Mexico Youth Risk and Resiliency Survey (NM YRRS) is part of the nationwide CDC Youth Risk Behavior Surveillance System. The NM YRRS is administered to high school students in odd-numbered years and uses two discrete population-based sampling designs. A smaller sample provides data representative of the state and a larger sample furnishes data on specific populations not well represented in the smaller state sample. In 2013, data from 5451 students were obtained for the statewide sample and data from 19,080 students were obtained for the larger sample. A minority of students, 26.1 %, were White, non-Hispanic. Hispanics comprised 59.2 % of the population, African Americans, 0.9 %, and other races/ethnicities, 13.7 %. Half (48.8 %) of students were female [58]. Suicidality rates among NM students far exceeded national averages; 15.6 % seriously considered suicide, 13.7 % planned an attempt, and almost 1 in 10 (9.4 %) attempted it in the last year [59].

The 2013 NM YRRS was the first to include questions about same-sex sexual contact and sexual identity of NM high school students, which enables the NM Department of Health and schools to better understand the health and safety risks of students. Almost 11 % of the sample identified as LGB, with 3.0 % identifying as lesbian or gay and 7.5 % as bisexual [60]. In NM, LGB students experienced a relative lack of safety at schools: 35.5 % reported being bullied on school property in the last year, compared with 17.0 % of their straight peers; 17.3 % skipped school because of safety concerns, compared with 5.6 % of their peers [61]. Suicidality rates among NM LGB students were strikingly high with 38.3 % considering suicide, 35.4 % reporting making a suicide plan, and 28.5 % attempting suicide in the past year [61].

### Study design

This 5-year cluster randomized trial will include 40 NM high schools that employ a nurse on staff (*n* = 64). The schools will be randomized into the RLAS intervention condition (IC; *n* = 20) or the control condition (CC; *n* = 20). Cluster randomization was chosen because the intervention will be delivered at the school level. Schools will be eligible if they express interest in the full intervention and the school nurse and a lead administrator agree to participate and support the project. To minimize confounding effects, eligible schools will share the following characteristics: (1) public high school; (2) school nurse willingness to convene an IRT if randomized to the IC group; and (3) informed written consent from the school nurse to participate for four years. Additional file 1 contains confirmation of ethical approval.

From the IC schools, there will be three study populations: (1) school nurses; (2) school administrators; and (3) IRT members. From CC schools, there will be two study populations: (1) school nurses and (2) school administrators. School nurses and IRTs in the IC schools will receive innovation supports to encourage greater implementation of the six EB strategies (see Table 1). Nurses in the IC will recruit IRT members (4–6 people) willing to work with them on implementing the six EB strategies. These nurse champions will function as coordinators who keep the IRTs motivated, cohesive, and focused on goals; gain administrative buy-in during the Preparation Phase; and facilitate IRT planning in the Implementation Phase.

### Aim 1: utilize the DAP to enable specially-trained school nurse champions and IRTs to implement and sustain EB strategies to address the needs of LGBTQ high school students

#### Exploration phase

For the exploration phase, the DAP centers on recruiting, enrolling, and randomizing schools, followed by initial assessments of system, provider, and client data to identify school needs, strengths, barriers, and readiness to implement the EB strategies. Results of the initial assessments will be shared with the IC schools specifically via comprehensive summaries to be vetted by the IRTs and integrated into their respective planning for implementation.

##### Qualitative data collection

Readiness interviews will consist of open-ended questions that will be asked of IC stakeholders at the system, provider, and client levels. These include 20 administrators, 20 school nurses, and 20 individuals randomly selected from the IRTs (*n* = 60). Interviews will explore if the necessary conditions exist for implementation and sustained use of the EB strategies and how best to optimize them. Questions will center on attitudes toward, access to, and availability of school and community supports for (a) suicide prevention, (b) LGBTQ youth, and (c) school nursing staff. Verbal and written information regarding school policies and practices pertinent to EB strategies will be collected. Specific questions will focus on transgender and intersex youth, i.e., perceptions and knowledge related to their risks for suicide, substance use, bullying, and truancy within school settings, and extant support. Finally, interviews will address pragmatic concerns (e.g., staffing and resources) and organizational factors (e.g., leadership and supportive culture/climate) [43, 47, 48, 62]. Results of these first assessments will inform IRT efforts to build implementation capacity and subsequent plans to implement and sustain EB strategies. For comparison purposes, a complementary set of interviews will be completed with the 20 nurses and the 20 administrators in the CC schools.

##### Qualitative data analysis

All qualitative interviews will be digitally recorded and professionally transcribed. Hand written notes will be organized according to a standard format, with information on date, time, and length of the interaction, setting, and participants involved. Typed notes and transcripts will be imported into a password protected NVivo database for efficient organization and analysis via iterative readings or codings [63]. Responses will first be analyzed by “open coding” to discover themes, ideas, and issues; a coded index of topics addressed in the data will next be developed. “Focused coding” will then be used to determine which topics arise often and which represent unusual or particular concerns [64, 65]. NVivo search functions can quickly locate and cross-reference statements of interest from participants (e.g., all text coded with “bullying at school,” “nonjudgmental teachers,” and “implementation barriers”) to examine relationships in the data within and across participant groups (e.g., school nurse, administrator, and IRT member).

Interview findings will be triangulated across participant types, creating a matrix (a) describing specific themes related to key study issues outlined in the EPIS model, and (b) supporting data by participant type. A side-by-side comparison of the various perspectives will be used to identify points of convergence and divergence in all participant statements related to the themes under consideration. In this staged approach to analysis, sets of notes and transcripts will be coded and detailed memos that describe and link codes to each theme and issue will be created. Discrepancies in coding and analysis will be identified during this process and resolved during regular team meetings.

##### Quantitative data collection

The 40 administrators and 40 school nurses in both the IC and CC schools will complete the first of an annual 30-min web-based survey that assesses organizational characteristics of schools and administrator/nurse work attitudes.

*Measures*. Demographics include age, gender, sexual orientation, race/ethnicity, education, employment, experience, geographical area of work, and activities performed in schools. Attitudes toward sexual and gender minorities will be assessed through two validated and reliable instruments, the *Attitudes Toward Lesbians and Gays Scale *[66] and the *Attitudes Toward Transgender Individuals Scale *[67]. Organizational culture and climate will be assessed via the *Organizational Social Context Scale*, which has good psychometric properties with Cronbach’s alphas ranging from 0.78 to 0.94 [42]. Climate includes three domains that affect use of EB strategies: (1) stress; (2) engagement; and (3) functionality. Culture includes (1) proficiency (responsiveness, competence) and (2) resistance (apathy, suppression). Work attitudes consist of two measures, *job satisfaction* (ten items) and *organizational commitment* (13 items), with good reliability and validity [68–70]. Attitudes toward adopting EB strategies will be assessed using the *Evidence-Based Practice Attitude Scale*, which examines: (1) intuitive appeal of EB practices; (2) likelihood of adopting if required; (3) openness; and (4) perceived divergence between EB practices and current practice [71]. Leadership will be measured via the *Multifactor Leadership Questionnaire (MLQ) *[72]. The MLQ assesses transformational leadership, transactional leadership, passive/avoidant leadership (laissez-faire), and perceived outcomes of leadership (extra effort, effectiveness, satisfaction). The MLQ has good psychometric properties with Cronbach’s alphas ranging from 0.76 to 0.90 [41, 69]. School-community linkages will be measured through an LGBTQ-specific modification of the *Collaborating with Community Subscale*, an eight-item tool based on the Measure of School, Family, and Community Partnerships [73]. This tool rates school partnership practices for integrating resources and services from the community to strengthen school programs, learning, and well-being for LGBTQ students on a scale of 1 (not occurring) to 5 (extensively occurring) and offers insight into improvement areas and future directions. An adapted *Assessment of* *Climate Embedding Mechanisms* *(ACEM)* measure will elicit Likert scale ratings on how frequently each EB strategy is used, EB strategy adherence, and school leadership support [43]. In year 1, it will be completed by nurses and administrators to serve as a baseline assessment for each school.

*School administrative data*. Publicly-available administrative data will be used to assess faculty and staff retention, school truancy, graduation rates, drop-out rates, extracurricular student activities, and overall student characteristics, e.g., socioeconomic status and race/ethnicity, at participating schools.

##### Quantitative data analysis

Quantitative analysis of the web-based surveys will involve descriptive analyses, aggregated at the school level, for each subscale in the organizational web-based survey. Data will be compared across schools to determine areas of relative strength and weakness regarding factors known to impact implementation. Data will be shared with the IC IRTs in the form of feedback reports comparing each school’s scores on each subscale to the average scores across other schools. The raw data, feedback, and recommended changes will help ensure that the IRTs recognize barriers that could affect implementation and will provide impetus for the IRTs to enhance deficit areas. For example, if the data reveal that a school has a defensive climate, an IRT can then determine ways to work under this constraint or, alternatively, to improve the climate. The analysis of school administrative data will also involve descriptive analyses of staffing, faculty/staff retention, truancy, graduation and drop-out rates, extracurricular student activities, and overall student characteristics, which will be compared across schools to identify potential confounders.

##### Feedback reports

After completing qualitative and quantitative analyses, the research team will prepare summaries that describe issues, ideas, and concerns raised by the school nurses, administrators, and IRTs at the IC schools, implementation readiness at the school and staff levels, and the encompassing school context. These summaries will include contextual detail for nuanced implementation planning through the DAP and outline strengths and weaknesses in the school settings that may influence implementation and uptake of each of the EB strategies. No feedback will be provided to the CC schools until the final year of the project.

#### Preparation phase

The IRTs review data from the exploration phase in the preparation phase to determine (a) adaptations needed in the school context and its workforce to ensure uptake; and (b) how to accomplish such adaptations. The school-specific feedback reports made available to the IRTs will provide information on relative strengths and weaknesses and key areas for the IRTs to address when implementing the strategies. Each school nurse-led IRT will review their relevant report, analyzing the extent to which their schools possess the requisite conditions for implementing each strategy. The nurse champion will guide the IRT through a process for prioritizing and addressing recommendations [74], weighing each based on five dimensions (importance, cost, time, commitment, and feasibility). The IRT would then outline the steps needed to follow through with actions. If a potential action is deemed “infeasible” at that time, the nurse would encourage the IRT to consider more “workable,” short-term strategies to create conditions sufficient for implementation.

Coaching for IC schools begins in the preparation phase. Coaches will engage in monthly conference calls with each nurse-led IRT supplemented with bi-monthly visits to the IC schools to identify additional training needs and create Resource Guides and Referral Lists to support practices related to the EB strategies. The guides will contain written material about advancing EB strategies, and the lists will identify community health and mental health providers and resources for LGBTQ youth and their peers. Coaching staff have specialized expertise in team building, school climates, LGBTQ youth intervention, suicide prevention, and transgender populations. Their role is to improve school nurse and IRT performance through both training and technical assistance (including development and delivery of webinar content), enabling them to hone the knowledge and skills for meeting EB strategy implementation goals.

#### Implementation phase

Training with adaptation support begins in the implementation phase. Twenty nurse-led IRTs will annually implement or strengthen a minimum of two of the six EB strategies per year, building on lessons accumulated over time. Ongoing assessment will occur to optimize local adaptations to enhance implementation. Fidelity and adherence data will be collected and provided as feedback to coaches and IRTs.

The overall training will emphasize explicit inclusion and discussion of adaptation, i.e., why and what to adapt, when to seek guidance on adaptation, and how to use the coaches and IRT. Further augmentation may be accomplished by including materials and training in response to unique school level barriers or populations (e.g., lack of LGBTQ-inclusive anti-bullying policies or negative teacher attitudes about LGBTQ youth). School nurses will be provided with a one-day in-person training that coincides with an annual conference sponsored by the NM School Nurses Association. The training will cover leadership that supports effective EB strategy implementation. To prepare nurses to be strong change agents in schools and to negotiate interdisciplinary IRTs, adapted modules (e.g. development of positive IRT climates and organizational support for EB strategies) of an implementation leadership curriculum that features traditional didactics and role-play activities are included in the training. The modules will be reinforced through regularly scheduled coaching and annual in-person booster trainings for school nurses in the implementation phase [75]. The IRTs will also benefit from eight 1-h webinars on LGBTQ people, youth suicide prevention, and school safety and supports. Trainees will receive monetary incentives or continuing medical education (CME) credits.

Coaching in deployment of the strategies will occur during the implementation phase. Coaches will continue to organize monthly conference calls with each IRT and conduct bi-monthly visits to IC schools to provide technical assistance. These efforts are intended to enhance the likelihood that EB strategies are implemented in the IC schools, and will contribute to adjustments to school-specific implementation protocols, if warranted. Coaches will compile logs of interactions with nurses and IRTs, which will be analyzed for process evaluation.

#### Sustainment phase

During the sustainment phase, the IRT analyzes implementation successes and challenges, focusing on school nurse and member satisfaction, adherence and fidelity to action plans, use of EB strategies, and the NM YRRS outcomes over time.

### Aim 2: conduct a cluster randomized controlled trial to assess whether LGB youth and peers in RLAS intervention schools report reduced suicidality, depression, substance use, and bullying, and increased safety compared to control schools

A cluster randomized controlled trial will assess whether LGBTQ students and their peers in RLAS intervention schools report reductions in suicidality, depression, substance use, bullying, and truancy related to safety concerns compared to those in usual care schools. Additional file 2 provides a study-specific checklist of information to include when reporting a cluster randomized trial. Additional file 3 contains a flow diagram of the phases of the randomized trial for the IC and CC schools. Randomization will take place in the exploration phase, as noted previously. Mahalanobis distance metric matching will be used to create pairs of similar schools [76], where one school in each pair will be randomly assigned to the IC.

The 2015 NM YRRS will offer baseline data for all schools and the 2017 and 2019 NM YRRS will yield follow-up data for the implementation and sustainment phases. From each school an average of 150 students complete the NM YRRS, for a sample size of 6000 per survey administration. Demographic data include gender, age, race/ethnicity, and sexual identity. Primary outcome measures assess suicide-related outcomes (“During the past 12 months, did you ever seriously consider attempting suicide?” “During the past 12 months, did you make a plan about how you would attempt suicide?” “During the past 12 months, how many times did you actually attempt suicide?”). Secondary measures assess depression (“During the past 12 months, did you ever feel so sad or hopeless almost every day for two weeks or more in a row that you stopped doing some usual activities?”), substance use (“During the past 30 days, on how many days did you have at least one drink of alcohol?” “During the past 30 days, how many times did you use marijuana?”), bullying (“During the past 12 months, have you ever been bullied on school property?”), and school safety (“During the past 30 days, on how many days did you not go to school because you felt you would be unsafe at school or on your way to or from school?”).

Analysis will examine five hypotheses using NM YRRS data: (1) IC schools will have a greater reduction in suicide-related outcomes than CC schools; (2) IC schools will have a greater reduction in depression than CC schools; (3) IC schools will have a greater reduction in substance use outcomes than CC schools; (4) IC schools will have a greater reduction in bullying than CC schools; and (5) IC schools will have a greater improvement in school safety than CC schools. Each analysis assesses these effects for the entire population and whether they are more pronounced for LGB students. The five outcomes predicted in hypotheses 1–5 will likely be more pronounced for LGB students, relative to other students. As the NM YRRS does not include items addressing gender identity, interviews and focus groups with school nurses, administrators, and IRT members will elucidate perceived impacts on transgender and intersex youth.

#### Data analysis

Students are nested within school by time period groups (e.g., 2015 observations of one school), and these repeated observations of a school are each nested within one school. Hence, all quantitative models examining hypotheses will use multilevel modeling (i.e., hierarchical linear modelling) to conservatively adjust estimates for variability arising in outcomes due to student (level 1) nesting within school by time groups (level 2) and these repeated observations at the school level being nested within schools (level 3). Thus, all models will be run as random intercept regressions. Sampling weight and design information will be used to appropriately weight cases in analyses via multilevel pseudo maximum likelihood methods [77, 78] and Mplus^©^ [79]. Models for continuous variables will be run assuming normally distributed, continuous outcomes (unless descriptive analyses indicate zero-inflated Poisson models for count outcomes). Binary outcomes will be treated as having a binomial distribution and use a logit link function. Regression models (one for each measured outcome in all hypotheses) will regress the outcome on a dummy contrast representing study condition; a dummy contrast representing LGB; two contrasts representing linear and quadratic, i.e., u-shaped, change; the orthogonal interactions between condition and change contrasts; the orthogonal interactions between LGB, condition, and change contrasts; and all other component interactions not mentioned. These models represent whether (a) there was differential change as a function of condition and (b) whether this differential change was more pronounced among LGB students.

### Aim 3: examine the individual, school, and community factors influencing implementation and outcomes

For the RLAS process evaluation, school health policies, practices, and community linkages, and the status of EB strategies will be assessed and compared across IC and CC groups. This will be done annually each spring via semi-structured interviews with all school administrators (*n* = 40) and nurses (*n* = 40) in years 1–4. To assess individual and school influences, the web-based surveys will be repeated with IC and CC groups each year. All qualitative interviews and surveys will follow the data collection and analysis procedures used in the preparation phase. Using these same qualitative analysis procedures, the content of coaching logs that describe in-depth implementation issues requiring technical assistance in the IC schools over the intervention period will also be assessed. Analysis of the web-based surveys aggregated across IC and CC schools will help identify and compare differences in the organizational characteristics of schools and administrator/nurse work attitudes over time.

#### Data collection and analysis

Many schools may already utilize one or more EB strategy; yet use of all six in accordance with CDC specifications is uncommon [40]. There is also a need to assess potential diffusion of the RLAS to CC schools. The EB strategy implementation in all 40 schools will be tracked using the adapted ACEM incorporated into the annual web-based surveys completed by nurses and administrators [43]. This instrument will yield data on frequency of EB strategy usage, EB strategy adherence, and school leadership support. These data will be supplemented with descriptive information from qualitative interviews concerning the extent to which the RLAS may stimulate changes in implementation, diffusion, and overall implementation status of each EB strategy at each school.

Implementation progress and fidelity for each EB strategy in IC schools will be determined via a modified version of the *Stages of Implementation Completion* (SIC) checklist, an eight-stage measure that will assess progress for phases of pre-implementation (exploration/preparation), implementation, and sustainment using activity completion dates and duration of activities and does not require additional effort from the school nurse champions and IRTs beyond participating in the usual implementation process [80]. The research team will complete the SIC by coding data from interviews, surveys, action plans, and coaching logs concerning markers or milestones. These data sources offer insight into individual, school, and community factors affecting progress. The SIC will be adapted to capture key stages of implementation progress for the six EB strategies. These may include the following: (1) engagement; (2) consideration of feasibility; (3) readiness planning; (4) staff trained; (5) fidelity monitoring process in place; (6) implementation begins; (7) ongoing implementation, coaching, and feedback; and (8) competency. The SIC scores will indicate the level of RLAS implementation per IC school over time, allowing categorization of the IC schools into “low” versus “high” implementation sites. These categories will be used to organize analyses of qualitative data to determine characteristics shared among IC schools assigned particular scores and to describe common barriers and facilitators that may have affected their implementation progress.

During the implementation and sustainment phases, the research team will conduct annual focus groups with the 20 IRTs to document their common and particular experiences identifying and advancing adaptation supports to facilitate use of EB strategies and their overall progress and satisfaction with the DAP. Each focus group will consist of at least five IRT members (including the school nurse), take place on site when IRTs regularly meet, and last between 90 to 120 min. Members will complete demographic forms (recording age, gender, sexual orientation, race/ethnicity, education, employment, socioeconomic status, religion, etc.) and a 10-min survey on individual perceptions and experiences related to the IRTs and the DAP. The focus group guides will consist of eight to ten open-ended questions. Participants will be asked to reflect on satisfaction, EB strategy implementation (including contextual relevance, feasibility, strategies to overcome barriers, and processes for forging and maintaining local linkages with health, mental health, and LGBTQ resources), and perceived outcomes on students, schools, and communities. These collective discussions [81, 82] will illuminate factors influencing the ability of IRTs to maintain their original action plans, reasons for possible divergence from these plans (e.g., community resistance) and subsequent adaptations (community education), and provide insight into possible changes to school climate, safety, support, and minority stress for LGBTQ students in IC schools. Using iterative coding, responses on perceived strengths, weaknesses, adaptations, and needed improvements will be clustered.

Convergences and divergences in quantitative and qualitative data will be identified [83–86]. Analyses of survey, NM YRRS, ACEM, and SIC data will be summarized, and findings from different data sources will be compared to create a complete picture of DAP and EB strategy implementation in the IC schools over time. Results will be integrated through an inclusive process that values the input of all stakeholders (nurses, administrators, and IRT members). Data will be merged by the following: (a) linking qualitative and quantitative databases; and (b) embedding one within the other so that each plays a supportive role for the other. Results of each dataset will be placed side-by-side to examine: (1) *convergence* (do results provide the same answer to the same question, e.g., do interview data concur with NM YRRS data regarding impact of RLAS on suicide risk among LGBTQ students?); (2) *expansion* (are unanticipated findings of one dataset explained by another, e.g., can web-based survey data that suggest a disempowering school climate be explained by qualitative interview data?); and (3) *complementarity* (does embedding results of the qualitative analysis in the quantitative dataset help contextualize results, e.g., does it explain variability represented by confidence intervals or variance estimates in statistical analyses on “bullying on school grounds” and “fear-based bullying”?). Triangulated results will be shared annually with IRTs and schools.

## Discussion

The RLAS keeps with national priorities to (a) improve school-based services for pediatric populations, (b) focus on LGBTQ youth mental health, and (c) revolutionize the role of nurses in U.S. healthcare. In addition to LGBTQ youth suicide, the conceptual framework and methods for this novel nurse-led intervention are applicable to addressing the health-related concerns of other pediatric populations in schools as well. Through its collaborative processes to refine, improve, and sustain EB strategies in these systems, the RLAS represents an innovative contribution to implementation science that also addresses a pressing public health challenge.

## Additional files

Additional file 1: Confirmation of ethical approval. (PDF 100 kb)
Additional file 2: CONSORT 2010 checklist of information to include when reporting a cluster randomized trial. (DOCX 27 kb)
Additional file 3: CONSORT 2010 flow diagram. (DOC 44 kb)

## Abbreviations

ACEM: Assessment of Climate Embedding Mechanisms
CC: Control condition
CDC: Centers for Disease Control and Prevention
DAP: Dynamic Adaptation Process
EB: Evidence-based
GSA: Gay-Straight Alliance
IC: Intervention condition
IRT: Implementation Resource Team
LGB: Lesbian, gay, and bisexual
LGBTQ: Lesbian, gay, bisexual, transgender, and queer or questioning
MLQ: Multifactor Leadership Questionnaire
NM: New Mexico
RLAS: Implementing School Nursing Strategies to Reduce LGBTQ Adolescent Suicide
SIC: Stages of Implementation Completion
YRRS: Youth Risk and Resiliency Survey
