# Supplementary material for: Implementing school nursing strategies to reduce LGBTQ adolescent suicide: a randomized cluster trial study protocol
Source: Implement Sci. 2016 Oct 22;11:145. doi: 10.1186/s13012-016-0507-2 (PMC5075193; doi:10.1186/s13012-016-0507-2)
Supplement: Additional file 1: — Confirmation of ethical approval. (PDF 100 kb) [file 13012_2016_507_MOESM1_ESM.pdf]

PIRE

TO:	Cathleen Willging, PhD
FROM:	Elysia Oudemans<br>Office of Research Integrity and Compliance
DATE:	June 17, 2016
PROJECT TITLE:	Implementing School Nursing Strategies to Reduce LGBTI Adolescent Suicide
IRBNET ID:	787984-3
PROJECT CODE #:	
ACTION:	APPROVED
APPROVAL DATE:	June 8, 2016
EXPIRATION DATE:	June 8, 2017
SUBMISSION TYPE:	Response/Follow-Up
REVIEW TYPE:	Expedited Review

Thank you for your Response/Follow-Up submission for this project. Pursuant to 45 CFR 46, Pacific Institute's IRB #3 has APPROVED your submission. This IRB approval expires on June 8, 2017.

This approval is based on an appropriate risk/benefit ratio and a project design that minimizes the risks to research participants. All research must be conducted in accordance with this approved submission. Please note that any revision to approved protocols or materials must be approved by this IRB prior to implementation.

Unless the IRB has granted a Waiver of Informed Consent, Federal regulations require that investigators obtain the informed consent of subjects (or a legally authorized representative) in order to enroll them in the research. Please remember that informed consent is a process that begins with a description of the project and includes risks, benefits, and alternatives (if appropriate). Informed consent requires that participants understand their role in the research at the beginning of the project and throughout its duration.

In addition, unless the IRB has granted a Waiver of Documentation of Informed Consent, Federal regulations require investigators to obtain a signed consent form from each subject, and the person signing must be given a copy of the consent document.

All unanticipated problems involving risks to subjects or others must be reported promptly to PIRE's Office of Research Integrity and Compliance. All non-compliance issues or complaints regarding this project must also be reported promptly to this office.

When the project ends, you are required to submit a Closure/Final Report to the IRB. Please note that research records must be retained for a minimum of three years after the completion of the project. The funder or other entities may require longer retention.

Generated on IRBNet

If you have any questions, please contact Elysia Oudemans at 301-755-2757 or oudemans@pire.org. Please include your project title and IRBNet ID number in all correspondence with this IRB and PIRE's Office of Research Integrity and Compliance.

Generated on IRBNet
