# Supplementary material for: Implementing school nursing strategies to reduce LGBTQ adolescent suicide: a randomized cluster trial study protocol
Source: Implement Sci. 2016 Oct 22;11:145. doi: 10.1186/s13012-016-0507-2 (PMC5075193; doi:10.1186/s13012-016-0507-2)
Supplement: Additional file 2: — CONSORT 2010 checklist of information to include when reporting a cluster randomized trial. (DOCX 27 kb) [file 13012_2016_507_MOESM2_ESM.docx]

Additional file 2: CONSORT 2010 checklist of information to include when reporting a cluster randomized trial

| Item | | Standard Checklist item | | Extension for cluster designs | |
| --- | --- | --- | --- | --- | --- |
| Title | | Identification as a randomized trial in the title | | Implementing School Nursing Strategies to Reduce LGBTQ Adolescent Suicide (RLAS): A Randomized Cluster Trial Study Protocol | |
| Background and objectives | | Scientific background and explanation of rationale | | Lesbian, gay, bisexual, transgender, and queer or questioning (LGBTQ) youth are at heightened risk for suicide, often as a result of chronic stress from stigmatization, prejudice, and discrimination that occurs in schools. LGBTQ youth with greater school connectedness and safety report lower suicidal ideation and attempts. This study examines a multilevel intervention within U.S. high schools to increase implementation of six evidence-based (EB) strategies to address the needs of LGBTQ students. Enactment of these strategies at the school level is hypothesized to improve school climate and safety for LGBTQ youth, as well as their cisgender, heterosexual peers. We utilize a cluster randomized design, with randomization occurring at the school level, given that our intervention is a school-wide initiative and data on implementation progress and student outcomes will be collected at the school level. | |
|  |  | Specific objectives or hypotheses | | 1. Utilize the Dynamic Adaptation Process (DAP) to enable specially-trained school nurse champions and Implementation Resource Teams (IRTs) to implement and sustain EB strategies at the cluster level (schools) to address the needs of LGBTQ high school students. 2. Conduct a cluster randomized controlled trial to assess whether lesbian, gay, and bisexual (LGB) youth and peers in RLAS intervention schools report reduced suicidality, depression, substance use, bullying, and increased safety compared to control schools. Individual level student accounts will be examined using multilevel modeling to account for the clustering effect of students within schools. 3. Examine the individual, school, and community factors influencing implementation and outcomes at the cluster level (schools). | |
| Methods | | | | | |
| Trial Design | | Description of trial design (such as parallel, factorial) including allocation ratio |  | | In this study, the cluster is defined as one high school with a nurse on staff. Forty clusters will be randomized into either the RLAS intervention condition (IC) or the control condition (CC). Intervention condition schools will receive training and coaching support to participate in the DAP to form IRTs tasked with implementing the six EB strategies. Qualitative data on implementation preparation, progress, and outcomes will be collected from school nurses and administrators in IC and CC schools via individual interviews and web-based surveys, and from IRTs in IC schools via focus groups. Supplemental data will derive from logs kept by RLAS coaches. Baseline and follow-up data on student outcomes for all schools will come from the 2015 (baseline) and 2017 and 2019 (follow-up) New Mexico Youth Risk and Resiliency Study (YRRS), which is administered to high school students as part of the nationwide Youth Risk Behavior Surveillance System. Analysis will examine five hypotheses using these data: (1) IC schools will have a greater reduction in suicide-related outcomes than CC schools; (2) IC schools will have a greater reduction in depression than CC schools; (3) IC schools will have a greater reduction in substance-use outcomes than CC schools; (4) IC schools will have a greater reduction in bullying than CC schools; and (5) IC schools will have a greater improvement in school safety than CC schools. Additional analysis will examine implementation progress and fidelity in IC schools. Findings from all data sources will be compared to create a complete picture of EB strategy implementation in IC schools over time. |
| Participants | | Eligibility criteria for participants | | Eligibility criteria for clusters:   1. Public high school 2. School nurse willingness to convene an IRT if randomized to the IC group 3. Informed written consent from the school nurse to participate for four years   Eligibility criteria for IRT members:   1. Willing to work with school nurse to implement EB strategies | |
|  |  | Settings and locations where the data were collected | | Data will be collected during in-person interviews and focus groups on school grounds and by web-based surveys of school nurses and administrators. School data will be collected via the YRRS and obtained from the New Mexico Department of Health. | |
| Interventions | | The interventions for each group with sufficient details to allow replication, including how and when they were actually administered | | The RLAS intervention will be delivered at the cluster level. Following the DAP, school nurses in IC schools will convene IRTs and receive ongoing training and coaching support to adapt, plan, and implement the six EB strategies. The intervention will proceed in four phases:   1. **Exploration:** The research team recruits, enrolls, and randomizes schools, and conducts an initial assessment of system, provider, and client data to identify school needs, strengths, barriers, and readiness to implement EB strategies. 2. **Preparation:** The IRTs review data from Exploration Phase to determine adaptations to EB strategies needed in school context and workforce and how to accomplish adaptations; IRTs recommend actions to improve school performance on EB strategies; coaches provide monthly support to IRTs, including additional training and creation of Resource Guides and Referral Lists. 3. **Implementation:** The IRTs enact plans to implement or strengthen a minimum of two EB strategies per year; coaches provide ongoing training and adaptation support. 4. **Sustainment:** The IRTs analyze implementation successes and challenges. | |
| Outcomes | | Completely defined pre-specified primary and secondary outcome measures, including how and when they were assessed | | Outcome measures pertain to the cluster level and include:   1. The extent to which IC schools report reduced suicidality, depression, substance use, bullying, and increased safety compared to CC schools. 2. Implementation progress and fidelity for each EB strategy in IC schools. 3. Perceptions of implementation progress among IC school staff (administrators, school nurses, and IRT members) and satisfaction with the DAP. | |
| Sample size | | How sample size was determined | | Based on a priori power analyses, we anticipate adequate power (.80) to detect small effects (.10-.18) with a sample of 40 schools. From each school, a random sample of 150 students will complete the New Mexico Youth Risk and Resilience Survey (YRRS). Based on past analyses of high school data in the state of New Mexico, we anticipate an Intraclass Correlation Coefficient of approximately (.02-.04). | |
| Randomization | | | | | |
| Sequence generation | | Method used to generate the random allocation sequence | | Mahalanobis distance metric matching will be used to create pairs of similar schools, where one school in each pair is randomly assigned to RLAS. | |
| Implementation | | Who generated the random allocation sequence, who enrolled participants, and who assigned participants to interventions | | Study statisticians will implement the stratified random assignment. Mahalanobis distance will be calculated from school background characteristics related to outcomes of interest, such as current use of EB strategies, proportion of LGB students, and percent having attempted suicide. Schools will be sequentially sorted based on the Mahalanobis distance score (i.e., the population centroid), and sequential pairs of two will be taken and randomized to RLAS or control condition.  Schools will be enrolled by the study Principal Investigators. Written informed consent to participate will be obtained from school nurses and administrators prior to randomization. | |
| Blinding | | If done, who was blinded after assignment to interventions (for example, participants, care providers, those assessing outcomes) and how | | Not applicable | |
| Results |  |  | | | |
| Participant flow | | For each group, the numbers of participants who were randomly assigned, received intended treatment, and were analysed for the primary outcome  For each group, losses and exclusions after randomisation, together with reasons | | Please see Additional file 2: CONSORT 2010 flow diagram. | |
| Recruitment | | Dates defining the periods of recruitment and follow-up  Why the trial ended or was stopped | | Recruitment is ongoing. | |
| Harms | | All-important harms or unintended effects in each group | | There is a risk of a confidentiality breach should unauthorized individuals gain access to private information about the participants. Potential risks to participants from such breaches are primarily psychological and social. We will privately collect information regarding the gender identity and sexual orientation of participants, which could lead to embarrassment if disclosed in a community setting. The research team will avoid asking questions specific to an individual’s LGBTQ status during in-person data collection events; and coaches will avoid asking questions during coaching sessions that are specific to an individual participant’s status.  All researchers will be trained in confidentiality protection and all data will be maintained according to institute policies. | |
| Other Information | |  | | | |
| Registration | | Registration number and name of trial registry | | ClinicalTrials.gov (Record 0838.01.01) | |
| Funding | | Sources of funding and other support (such as supply of drugs), role of funders | | This study is supported by Eunice Kennedy Shriver National Institute of Child Health and Human Development Grant R01HD083399 (Principal Investigators: Mary M. Ramos and Cathleen E. Willging). | |
