# Supplementary material for: Implementing school nursing strategies to reduce LGBTQ adolescent suicide: a randomized cluster trial study protocol
Source: Implement Sci. 2016 Oct 22;11:145. doi: 10.1186/s13012-016-0507-2 (PMC5075193; doi:10.1186/s13012-016-0507-2)
Supplement: Additional file 3: — CONSORT 2010 flow diagram. (DOC 44 kb) [file 13012_2016_507_MOESM3_ESM.doc]

Schools assessed for eligibility (*n*=X)

Excluded (*n*=X)

- No full time school nurse (*n*= X)
- No baseline Youth Risk and Resilience Survey (YRRS) data (*n*=X)
- Declined to participate (*n*=X)
- Other reasons (*n*=X)

Schools randomized to the intervention condition (IC; *n*=20)

Fall 2015 baseline YRRS analyses

- Total IC schools (*n*=X)
- Total IC students (*n*=X)
  Excluded from analysis (*n*=X)
  - Reasons for exclusion

Fall 2016-Spring 2017

- Implementation resource team formation
- Training and coaching in first 2 strategies

Randomized (*n*=40)

Lost to follow-up (n=X)

- School did not participate in YRRS (*n*=X)
- Loss of support from school (*n*=X)

Fall 2017 YRRS analyses

- Total IC schools (*n*=X)
- Total IC students (*n*=X)
  Excluded from analysis (*n*=X)
  - Reasons for exclusion

Lost to follow-up (n=X)

- School did not participate in YRRS (*n*=X)
- Loss of support from school (*n*=X)

2019 YRRS analyses

- Total IC schools (*n*=X)
- Total IC students (*n*=X)
  Excluded from analysis (*n*=X)
  - Reasons for exclusion

Fall 2017-Spring 2018

- Training and coaching in second 2 strategies

Fall 2018-Spring 2019

- Training and coaching in last 2 strategies

Schools randomized to the control condition (CC; *n*= 20)

Fall 2015 baseline YRRS analyses

- Total CC schools (*n*=20)
- Total CC students (*n*=X)
  Excluded from analysis (*n*=X)
  - Reasons for exclusion

Lost to follow-up (n=X)

- School did not participate in YRRS (*n*=X)
- Loss of support from school (*n*=X)

Fall 2017 Baseline YRRS analyses

- Total CC schools (*n*=X)
- Total CC students (*n*=X)
  Excluded from analysis (*n*=X)
  - Reasons for exclusion

Lost to follow-up (n=X)

- School did not participate in YRRS (*n*=X)
- Loss of support from school0(*n*=X)

2019 YRRS analyses

- Total CC schools (*n*=X)
- Total CC students (*n*=X)
  Excluded from analysis (*n*=X)
  - Reasons for exclusion

**Additional file 2** CONSORT 2010 flow diagram.
